# Hst3p, a histone deacetylase, promotes maintenance of *Saccharomyces cerevisiae* chromosome III lacking efficient replication origins

**DOI:** 10.1007/s00438-015-1105-8

**Published:** 2015-08-29

**Authors:** Carmela Irene, James F. Theis, David Gresham, Patricia Soteropoulos, Carol S. Newlon

**Affiliations:** Department of Microbiology, Biochemistry and Molecular Genetics, Rutgers New Jersey Medical School, ICPH, 225 Warren St., Newark, NJ 07101-1701 USA; Department of Biology, Center for Genomics and System Biology, New York University, 100 Washington Square East, New York, NY 10003 USA

**Keywords:** SNP analysis, Genome stability, DNA replication, Histone H3 K56 acetylation, Sirtuin

## Abstract

Long gaps between active replication origins probably occur frequently during chromosome replication, but little is known about how cells cope with them. To address this issue, we deleted replication origins from *S. cerevisiae* chromosome III to create chromosomes with long interorigin gaps and identified mutations that destabilize them [originless fragment maintenance (Ofm) mutations]. *ofm6*-*1* is an allele of *HST3*, a sirtuin that deacetylates histone H3K56Ac. Hst3p and Hst4p are closely related, but *hst4*Δ does not cause an Ofm phenotype. Expressing *HST4* under the control of the *HST3* promoter suppressed the Ofm phenotype of *hst3*Δ, indicating Hst4p, when expressed at the appropriate levels and/or at the correct time, can fully substitute for Hst3p in maintenance of ORIΔ chromosomes. H3K56Ac is the Hst3p substrate critical for chromosome maintenance. H3K56Ac-containing nucleosomes are preferentially assembled into chromatin behind replication forks. Deletion of the H3K56 acetylase and downstream chromatin assembly factors suppressed the Ofm phenotype of *hst3*, indicating that persistence of H3K56Ac-containing chromatin is deleterious for the maintenance of ORIΔ chromosomes, and experiments with synchronous cultures showed that it is replication of H3K56Ac-containing chromatin that causes chromosome loss. This work shows that while normal chromosomes can tolerate hyperacetylation of H3K56Ac, deacetylation of histone H3K56Ac by Hst3p is required for stable maintenance of a chromosome with a long interorigin gap. The Ofm phenotype is the first report of a chromosome instability phenotype of an *hst3* single mutant.

**Electronic supplementary material:**

The online version of this article (doi:10.1007/s00438-015-1105-8) contains supplementary material, which is available to authorized users.

## Introduction

**Fig. 1:**
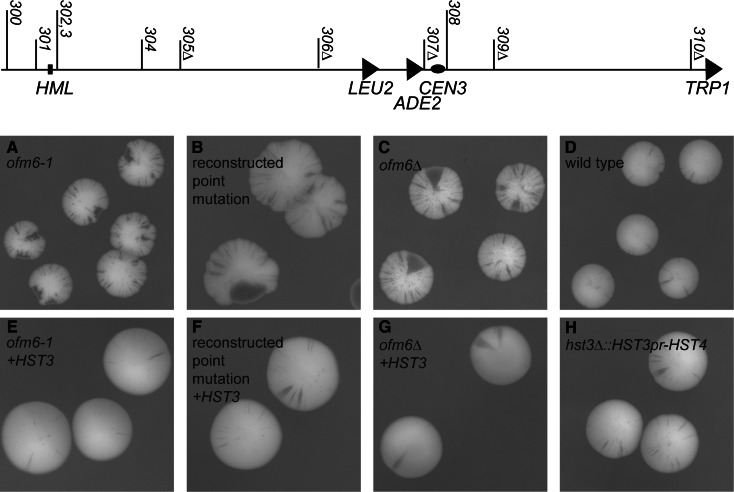
Ofm phenotype and complementation test. A schematic drawing of the 5ORIΔ-ΔR fragment of chromosome III is shown at the top. The positions of ARS elements are indicated by *numbers* above the *line*, with three digit numbers (e.g., *301*) indicating dormant origins still present on the fragment and three digit numbers followed by a “*triangle*” symbol (e.g., *305Δ*) indicating origin deletions. The *arrows* on the *line* represent the three selectable markers *LEU2*, *ADE2* and *TRP1*; the latter marks the position of the chromosome fragmentation that removed the right arm of the chromosome distal to the *ARS310* deletion. This fragment was introduced into both the wild-type (YKN15) and the *ofm6*-*1* mutant (YJT417) by chromoduction. After selection, chromoductants were plated for single colonies on medium containing limiting adenine, and incubated for 5 days at 30 °C. *Panels* **a**, **b** and **c** show colonies from three different *hst3* strains: the original *ofm6*-*1* isolate (YJT417), the reconstructed point mutant (YIC257) and the* hst3*Δ::*kanMX* mutant (YIC247), respectively. *Panel* **d** shows colony-sectoring phenotypes of the wild-type strain. Loss events are visualized as *red* sectors in *white* colonies. A complementation test was done by introducing the *HST3* gene into each of these mutants. A plasmid carrying the *HST3* ORF under the control of its own promoter was integrated into the non-essential *YGL119* *W* ORF by two-step gene replacement. Note that the *HST3* gene complements the colony-sectoring phenotype of all mutants: **e** *ofm6*-*1* (YIC275) **f** reconstructed point mutant (YIC271) and **g** *hst3*Δ (YIC273). *Panel* **d** shows the colony-sectoring phenotypes of a strain carrying the *HST4* ORF integrated in the *HST3* locus so that *HST4* expression is regulated by the *HST3* promoter plus the 3′ UTR (*hst3::HST3pr*-*HST4*)

The long, linear DNA molecules of eukaryotic chromosomes are replicated from multiple replication origins. In most eukaryotes, replication origins are inefficient, initiating replication in fewer than 30 % of cell cycles (Tuduri et al. 2010). Although regions of chromosomes replicate during reproducible times during S phase, initiation at individual origins is thought to be stochastic (Bechhoefer and Rhind 2012). These factors lead to the presence of long gaps between some active replication origins in most S phases. It is not known how cells cope with these long interorigin gaps. We created a derivative of *Saccharomyces cerevisiae* chromosome III from which we deleted the five most active replication origins (the 174-kb 5ORIΔ-ΔR fragment, see schematic diagram in Fig. 1), creating a long interorigin gap (Dershowitz et al. 2007). Even though the 5ORIΔ-ΔR fragment is duplicated and segregated properly in >99 % of cell divisions, it is sensitive to subtle perturbations in DNA replication, checkpoint surveillance, and chromatin structure (Theis et al. 2010). This sensitivity is likely created because replication initiates infrequently on this chromosome, causing replication forks to traverse much longer distances than normal. The maximum gap between origins mapped in *S. cerevisiae* is 90 kb, significantly below the gap size predicted for randomly distributed origins in intergenic regions. This finding suggests that the origin distribution has been at least in part determined to reduce the interorigin gaps to minimize the consequences of irreversible fork stalling (Newman et al. 2013). The ORI-deletion chromosome, creating a long unnatural gap between known origins, is a unique tool for uncovering pathways contributing to chromosome stability because the problems causing instability of the 5ORIΔ-ΔR fragment are likely to be experienced by wild-type chromosomes during the course of normal DNA replication when adjacent replication origins fail to initiate or converging forks stall between adjacent origins. To elucidate the mechanism(s) responsible for maintenance of the 5ORIΔ-ΔR fragment, we identified mutants that selectively destabilized it, but had little or no effect on the stability of the 0ORIΔ-ΔR fragment, which we named originless fragment maintenance (Ofm) mutants (Theis et al. 2007). In the study reported here, we demonstrate that *ofm6*-*1* is an allele of *HST3*, and show that the persistence during S phase of the acetylation of histone H3 lysine 56 normally removed by Hst3p and Hstp4 causes chromosome loss, likely by inhibition of replication fork movement.

Hst3p is a histone deacetylase (HDAC) that, together with the related HDAC encoded by *HST4*, removes acetyl groups from lysine 56 (K56) in the core domain of histone H3 (Celic et al. 2006; Maas et al. 2006). Hst3p and Hst4p belong to the family of NAD^+^-dependent HDACs called sirtuins, which are found in virtually all organisms. Budding yeast contain five sirtuins, Hst1p through Hst4p, and Sir2p, the founding member of the family (Brachmann et al. 1995). *HST3* and *HST4* have been implicated in maintenance of genome integrity in *S. cerevisiae* by the observation that simultaneous deletion of *HST3* and *HST4* causes defects in cell cycle progression, chromosome loss, spontaneous DNA damage, including gross chromosomal rearrangements (GCRs), base substitutions, small insertions and deletions, as well as acute sensitivity to genotoxic agents, and thermosensitivity. These phenotypes are all caused by constitutive H3 K56 acetylation (Celic et al. 2006; Kadyrova et al. 2013; Maas et al. 2006).

HST3 is controlled both at transcriptional and post-transcriptional levels. The protein is targeted for degradation after the phosphorylation of a multisite degron, and its turnover is increased in response to replication stress in a RAD53-dependent manner (Delgoshaie et al. 2014; Edenberg et al. 2014).

Histone proteins form the cores of nucleosomes, the fundamental units of chromatin. They undergo a variety of posttranslational modifications including phosphorylation, methylation, ubiquitination, sumoylation and acetylation. These modifications regulate several aspects of chromosome dynamics. Acetylation, in particular, occurs both on newly synthesized histone proteins, where it regulates nucleosome assembly and DNA repair, and on nucleosome-incorporated histones, where it regulates chromatin condensation, heterochromatin silencing and gene expression (reviewed by Shahbazian and Grunstein 2007). Acetyl groups are added to ε-amino groups of lysines by histone acetyltransferases (HATs) and removed by HDACs.

Acetylation was first observed within the N-terminal tails of the four histone proteins (Shahbazian and Grunstein 2007). However, at least two acetylation sites within the core domains of histones H3 and H4, lysine 56 in histone H3 (H3 K56) (Hyland et al. 2005; Masumoto et al. 2005; Ozdemir et al. 2005) and lysine 91 in histone H4 (Ye et al. 2005) are known. H3 K56 acetylation is found in Drosophila, mouse, rat and human cells (Das et al. 2009; Tjeertes et al. 2009; Xie et al. 2009; Yuan et al. 2009).

In *S. cerevisiae*, H3K56 acetylation occurs on newly synthesized H3 before its incorporation into chromatin by both replication-coupled and replication-independent mechanisms and is removed from nucleosomes during G2 and M phases (Masumoto et al. 2005). The H3K56Ac modification increases the efficiency of nucleosome assembly on DNA by increasing the binding affinities of the CAF-1 and Rtt106p chromatin assembly factors for histone H3 during DNA replication (Li et al. 2008), and is also important for the replication-independent turnover of nucleosomes (Kaplan et al. 2008).

A gene interaction analysis showed that *HST3* interacts with genes of an epistasis group that promotes genome stability and with deletions that perturb DNA replication. The *hst3* mutation showed positive genetic interactions with members of this epistasis group, including *asf1Δ*, *rtt109Δ*, *rtt101Δ*, *mms1Δ* and *mms22Δ* (Collins et al. 2007; Pan et al. 2006). The *RTT109*-encoded acetyltransferase is the predominant HAT for H3 K56 (Driscoll et al. 2007; Han et al. 2007; Tsubota et al. 2007). Rtt109p has been shown to form separate complexes with two histone chaperones Vps75p and Asf1p. *ASF1* deletion, but not *VPS75* deletion, completely abolishes H3 K56 acetylation in vivo (Tsubota et al. 2007). Rtt101p is a cullin that assembles a multi-subunit E3 ubiquitin ligase complex that preferentially binds and ubiquitinates histone H3 acetylated on lysine 56. Mms1p and Mms22p are adaptor proteins for substrate binding. Moreover, this ubiquitination weakens the Asf1 and H3–H4 interaction facilitating the transfer of the histone heterodimer to other histone chaperones, CAF-1 and Rtt106p, for nucleosome assembly (Han et al. 2013; Zaidi et al. 2008).

In this study, we found Hst3p in two separate screens for maintenance of originless chromosomes showing a link between chromosome stability—in particular DNA replication progression—and H3 K56 acetylation. Our evidence also showed that, despite difference in amino acid sequence and timing of expression, the closely related homolog *HST4* can complement the *hst3* mutation. We confirmed that the cycling of the H3K56Ac modification between G2/M and S phase is crucial for proper chromosome maintenance, and that the presence of H3K56Ac ahead of replication forks causes ORI∆ chromosome loss.

## Materials and methods

### Strains and plasmids

Yeast strains used in this study are listed in Table S1. All YKN10 and YKN15 derivatives are isogenic with YPH499 (Sikorski and Hieter 1989); the chromosome fragment donor strains are in the CF4-16B background (Dershowitz et al. 2007); the BY4741-derived strains are related to S288C (Brachmann et al. 1998). The strains used for *ofm6*-*1* linkage analysis were YJT371, harboring the *ofm6*-*1* mutation and BY4741 derivatives, each carrying a *kanMX*-marked deletion (Table S2). Deletions of both copies of the genes encoding histone H3 and histone H4 were introduced into the YPH499 background by crossing MSY421 (provided by MM Smith, Univ. of Virginia) to YJT3. The sole source of histones H3 and H4 in this *hht1Δ hhf1Δ, hht2Δ hhf2Δ* strain(YIC335) is the plasmid, pMS329 (*HHT1*-*HHF1*-*CEN4*-*URA3*) (Megee et al. 1990). Several strains were constructed by introducing PCR products carrying *kanMX*-marked deletions amplified from the Open Biosystems systematic deletion collection by transformation. 5ORIΔ-ΔR and 0ORIΔ-ΔR fragments were introduced into yeast strains by chromoduction as described by Theis et al. (2007).

Plasmids constructed for this study include the *ofm6*-*1* point mutation rescue plasmid, the *HST3::URA3* plasmid used for integration of *HST3* into the intergenic region adjacent to the *YGL119* *W* ORF, the* hst3*Δ::*HST3pr-HST4* construct used for expressing Hst4p under the control of Hst3p regulatory sequences, and the *TRP1 CEN6* plasmids expressing the histone H3 K56R and K56Q alleles used in plasmid shuffle experiments. Details of constructs are available upon request.

### Loss rate determinations

Fluctuation analyses were performed as described previously (Dershowitz and Newlon 1993). For the half-sectored colony assay, cells were plated on color assay medium and incubated for 5 days at 30 °C. Approximately 1000 total colonies were analyzed for each time point. Loss rates were calculated as the fraction of half-sectored colonies obtained in at least 4 independent experiments.

### Cell synchronization and FACS analysis

MAT**a** cells (1 × 10^7^cells/ml) were incubated for 3 h at 25 °C with 5–10 nM Nle-12 α-factor (Raths et al. 1988) (a gift from F. Naider, College of Staten Island, The City Univ. of New York), then released from G1 arrest by washing out α-factor and transferring the cell suspension to fresh YPD containing 0.1 mg/ml protease (Sigma-Aldrich). Cell cycle progression was monitored by Flow Cytometry with a LSR II four laser bench top Immunocytometry System (BD Biosciences) as described (Paulovich and Hartwell 1995).

### Microarray analysis

The Affymetrix GeneChip^®^*S. cerevisiae* Tiling 1.0R Array was used in this study. The probes on the array are 25-mer oligonucleotides tiled at an average resolution of 5 base pairs. This array design was previously used to locate SNPs and deletion breakpoints in the yeast genome (Gresham et al. 2006). DNA was isolated using QIAGEN Genomic-tip 500G according to the manufacturers protocol. DNA (10 μg) was digested to 50-bp fragments using 1U of DNase I (Amersham) and end-labeled with biotin using the GeneChip Double-Stranded DNA Terminal Labeling kit (Affymetrix). The biotin-labeled DNA was hybridized to the array for 16 h at 45 °C using a GeneChip Hybridization Oven 640. Washing and staining with Streptavidin–phycoerythrin were performed using the GeneChip Fluidics Station 450 and the FS450_0001 Fluidics Protocol. Images were acquired using the Affymetrix Scanner 3000 7G. The microarray data were analyzed using the SNPScanner algorithm as previously described (Gresham et al. 2006). We detected mutations in the mutant strain by comparing SNPs detected in the *ofm6*-*1* strain with SNPs detected in the isogenic wild-type strain. This was necessary as the wild-type strain used in this study differed substantially in sequence content from the S288C reference genome. We employed the identical heuristic cutoffs used to minimize false-positive SNP detection as described by Gresham et al. (2006).

### Analysis of replication intermediates

Analysis was performed as previously described (Theis and Newlon 2001).

## Results

### Classical genetic and genome-wide approaches to identify the *ofm6*-*1* mutation

Our initial goal in this study was to identify the gene harboring the recessive *ofm6*-*1* mutation (Theis et al. 2007). Ofm mutations were identified using a visual screen based on colony sectoring. The strains used for these screens are partially disomic for chromosome III, carrying a wild-type balancer chromosome III and the 5ORIΔ-ΔR fragment of chromosome III (Dershowitz et al. 2007), introduced by chromoduction (Ji et al. 1993). This chromosome fragment is genetically marked in a way that allows both its selection and the visualization of its loss in a colony-sectoring assay. Losses of the 5ORIΔ-ΔR fragment are visualized as red sectors in a white colony (Fig. 1).

We could not use the classical approach of complementation with a wild-type plasmid library to identify the mutation because, as we learned in our attempt to complement the *ofm14*-*1* mutation, the frequency of missegregation of the fragment, which produces white, non-sectoring colonies, was much greater than the probability of finding a complementing plasmid (Theis et al. 2007). Moreover, the *ofm6*-*1* strain was insensitive to UV and to several drugs [methyl methanesulfonate (MMS), hydroxyurea (HU), phleomycin and camptothecin] tested to identify a secondary phenotype for complementation; therefore, we pursued a different strategy. We searched genome wide for single nucleotide polymorphisms (SNPs), using a high-density Affymetrix yeast tiling microarray (Gresham et al. 2006). The *ofm6*-*1* mutant was compared to its wild-type parent, YKN10, to reveal SNPs unique to the *ofm6*-*1* mutant. A total of 244 candidate SNPs were identified in the mutant and were distributed as follows: 142 in ORFs, 43 in intergenic regions, 48 in repeated sequences (retrotransposons, *Y*′ elements, long terminal repeats, rDNA, ARS elements and telomeres) and 11 in introns and dubious ORFs.

Classical genetic mapping allowed us to reduce the number of candidate mutations that could account for the *ofm6*-*1* phenotype, as crosses to strains carrying mutations in genes involved in DNA replication revealed that *ofm6*-*1* is on chromosome XV (Table S2).

From our screen of the *S. cerevisiae* viable deletion collection for new Ofm mutants (Theis et al. 2010), we knew that strains carrying deletions of three adjacent ORFs near *CEN15*, *YOR024* *W*, *HST3* and *BUB3* showed a colony-sectoring phenotype. In the microarray analysis, we found that a SNP mapped at chromosome XV coordinate 378826, which is located in the *HST3* ORF (Figure S1), moreover, there were no SNPs in either *BUB3* or *YOR024* *W* in the *ofm6*-*1* mutant strain (Figure S2). Nucleotide sequence analysis of the *HST3* gene recovered from the *ofm6*-*1* strain confirmed the presence of the predicted SNP, which changes the Trp206 codon (UGG) into a stop codon (UAG).

### *ofm6*-*1* is an allele of *HST3*

To confirm that *ofm6*-*1* is an allele of *HST3,* we reconstructed the point mutation in a wild-type strain. This strain had a phenotype indistinguishable from that of the original *ofm6*-*1* mutant (Fig. 1a, b), confirming that the mutation identified in *hst3* caused the sectoring phenotype in the *ofm6*-*1* mutant.

We also moved the* hst3*Δ::*kanMX* deletion into the wild-type YKN15 strain background by one-step gene replacement. The resulting strain showed a colony-sectoring phenotype and a loss rate of the 5ORIΔ-ΔR fragment (1.2 ± 0.3 × 10^−2^) comparable to the original *ofm6*-*1* mutant (Theis et al. 2007), confirming the strong Ofm phenotype observed in the SGA strain background (Fig. 1c). We showed that the *HST3* wild-type gene, either on a plasmid or integrated into an ectopic locus in the genome, complemented *ofm6*-*1,* the reconstituted point mutant, and the ORF deletion (Fig. 1d–f). Finally, we found elevated H3K56Ac levels persisting in the G2 phase of the cell cycle in both *ofm6*-*1* and* hst3*Δ mutants (Figure S3), as others have previously shown (Celic et al. 2006).

Although the* hst4*Δ mutation alone does not cause an Ofm phenotype (data not shown), we were unable to isolate* hst3*Δ*hst4*Δ double mutant chromoductants carrying the 5ORIΔ-ΔR fragment, suggesting that Hst4p also contributes to maintenance of this fragment.

### Hst4p can substitute for Hst3p

*HST3* and *HST4* sequences are 26 % identical and 42 % similar overall, with 40 % identity in the core deacetylase domain (Brachmann et al. 1995). Hst3p differs from Hst4p in having a C-terminal extension of 107 amino acids. Expression patterns of Hst3p and Hst4p also differ, with maximal Hst3p expression occurring during G2/M and maximal Hst4p expression during M/G1 (Maas et al. 2006). To determine whether differences between Hst3p and Hst4p proteins or differences in their expression profiles account for the observation that *hst3* mutations, but not *hst4* mutations cause an Ofm phenotype, we tested whether the *HST4* ORF could complement the *hst3* colony-sectoring phenotype when placed under the control of *HST3* regulatory sequences by constructing strains in which Hst4p is expressed under control of both its normal regulatory sequences at its endogenous locus and the *HST3* upstream and downstream regulatory elements at the *HST3* locus. Four independent strains carrying the ORF swap showed a colony-sectoring phenotype comparable to a wild-type strain, indicating that expression of *HST4* from the *HST3* locus complements the* hst3*Δ colony-sectoring phenotype (Fig. 1d, h). We conclude that the two proteins are interchangeable and that Hst4p can fully substitute for its homolog Hst3p in the maintenance of the 5ORIΔ-ΔR fragment. This result indicates that either the timing or level of expression of Hst3p is responsible for its predominant role in 5ORIΔ-ΔR chromosome maintenance, not its differences in amino acid sequence from Hst4p.

### Role of the *RTT109*-*MMS22* acetylation pathway in the maintenance 5ORIΔ-ΔR chromosome

**Fig. 2:**
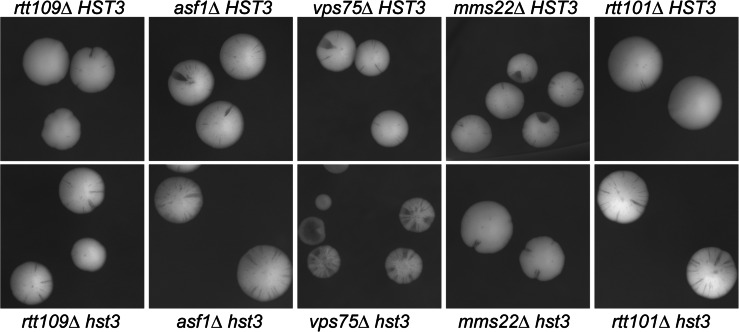
Deletion of components of the Rtt109p acetylation pathway suppresses the *hst3* colony-sectoring phenotype. The *panels* show colony-sectoring phenotypes of strains carrying* rtt109*Δ,* asf1*Δ,* vps75*Δ,* mms22*Δ and* rtt101*Δ in both *HST3* (*top panels* strains YIC263, YIC290, YIC302, YIC304, YIC266) and *hst3* strains (*bottom panels* strains YIC260, YIC296, YIC301, YIC306, YIC264)

To address a possible role for the *RTT109*-*MMS22* pathway (Collins et al. 2007) in 5ORIΔ-ΔR fragment maintenance, we determined the colony-sectoring phenotype of strains carrying deletions of genes in this pathway, both singly and in combination with an *hst3* allele. Analysis of the colony-sectoring phenotype showed that none of the single mutations tested caused an increase in the loss rate of the 5ORIΔ-ΔR fragment (Fig. 2). However, deletion of *ASF1* or *RTT109*, which are both required to acetylate H3K56, suppressed the colony-sectoring phenotype of *hst3* strains, while deletion of *VPS75*, which encodes another histone chaperone that interacts with Rtt109p, did not.

Rtt101p, Mms1p and Mms22p form a complex that preferentially ubiquitinates K56-acetylated histone H3, which facilitates the incorporation of H3K65Ac-containing nucleosomes into chromatin behind the replication fork. Deletion of *RTT101* or *MMS1* decreases the amount of H3K56Ac in chromatin (Han et al. 2013). Deletion of either *RTT101* or *MMS22* suppressed the *hst3* phenotype, suggesting that the persistence of H3K56Ac in chromatin causes the Ofm phenotype.

While others have shown that lack of Rtt101 suppresses the temperature- and DNA damage-sensitive phenotypes of the double *hst3hst4* mutant, we directly evaluated a chromosome loss phenotype to show that the same suppression occurs in the *hst3* mutant.

### H3K56Ac is the Hst3p substrate critical for 5ORIΔ-ΔR chromosome maintenance

**Fig. 3:**
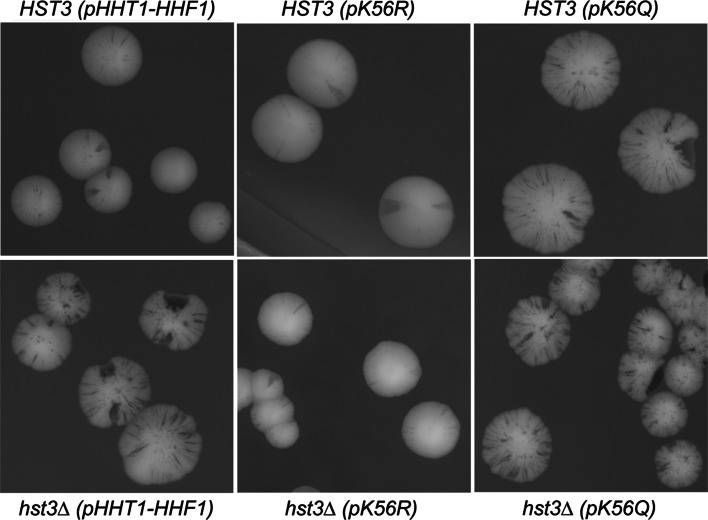
H3K56Ac is the critical target of *HST3* deacetylase. All the strains shown carry deletions of both copies of the genes encoding histones H3 and H4 (*hht1*Δ-*hhf1*Δ and* hht2*Δ-*hhf2*Δ). The sole source of histones H3 and H4 in these strains is a plasmid carrying wild-type histone genes p*HHT1*-*HHF1* (strains YIC335, YIC349), a plasmid carrying an H3 K56R substitution p*hht2K56R*-*HHF2* (YIC339, YIC351), or a plasmid carrying an H3 K56Q substitution p*hht2K56Q*-*HHF2* (YIC345, YIC350). The *top row* shows colonies of a wild-type *HST3* strain transformed with each of the plasmids. The *bottom row* shows colonies of the* hst3*Δ strains transformed with each of the plasmids

The fact that *rtt109, asf1, rtt101* and *mms22* deletions suppressed the *hst3* sectoring phenotype suggests that H3K56Ac is a relevant Hst3p substrate for 5ORIΔ-ΔR fragment maintenance. However, it is possible that acetylation of a different, as yet unknown, Rtt109p target contributes to this phenotype. To directly test the relevance of H3 K56 deacetylation in 5ORIΔ-ΔR fragment stability, we constructed a set of yeast strains expressing derivatives of histone H3 in which K56 was changed either to glutamine (K56Q) or arginine (K56R) as the sole source of histone H3, and then we followed 5ORIΔ-ΔR fragment stability by monitoring the colony-sectoring phenotype in both *HST3* and* hst3*Δ strains. If H3K56Ac is the critical Hst3p substrate for 5ORIΔ-ΔR fragment stability, then the strains expressing only H3K56Q, which mimics constitutive acetylation, should recapitulate the* hst3*Δ Ofm phenotype in the *HST3* strain. In addition, H3K56Q expression should not exacerbate the *hst3* phenotype, because H3 K56Q mimics constitutive acetylation. On the other hand, the instability of the 5ORIΔ-ΔR fragment should be suppressed in *hst3* strains expressing only H3 K56R, which cannot be acetylated. In addition, the phenotype of the *HST3* strain should not be affected by this substitution, because we have shown that* rtt109*Δ strains, which lack the HAT, do not exhibit an elevated colony-sectoring phenotype (Fig. 2). The results validated the expectations. Only the plasmid expressing H3 K56Q caused a strong colony-sectoring phenotype in an *HST3* strain (Fig. 3b). Similarly, strong sectoring phenotypes were apparent in* hst3*Δ transformants expressing either the wild-type histone H3 or the H3 K56Q allele (Fig. 3d, e). Expression of the K56R allele suppressed the colony-sectoring phenotype of the* hst3*Δ strain, but it had no effect in a wild-type *HST3* strain (Fig. 3c, f). These results indicate that Hst3p deacetylation of H3K56Ac is crucial for 5ORIΔ-ΔR fragment stability. They also exclude mechanisms dependent on other deacetylase target(s) or deacetylase-independent activities of Hst3p.

### The presence of H3K56Ac in the second cell cycle following inhibition of sirtuins causes 5ORIΔ-ΔR chromosome destabilization

**Fig. 4:**
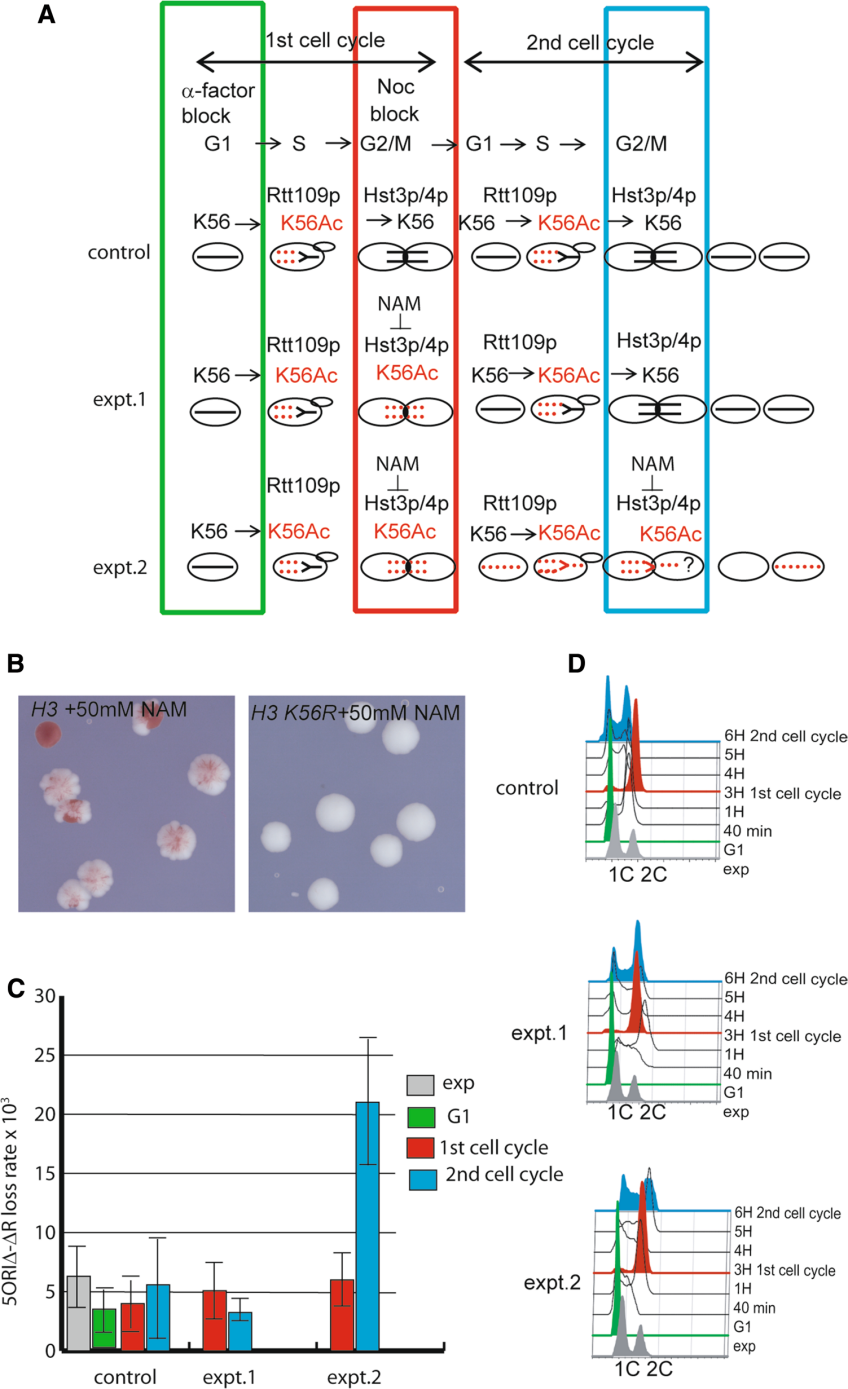
H3 K56 Hyperacetylation and DNA replication. **a** The *top row* shows an outline of the experiment with *arrows* indicating progression to consecutive steps of the cell cycle, G1, S and G2/M. This experiment was done with a *HST3* strain (YKN15). The three schematic drawings below represent the H3 K56 acetylation patterns expected in the three experimental conditions tested. A single chromosome is shown as an example. *Solid black lines* indicate chromatin with non-acetylated H3 K56; *dotted red lines* indicate chromatin with acetylated H3 K56. *Colored boxes* indicate the points at which samples were taken for plating (*Panel* ***c***) and FACS analysis (*Panel* **d**). In the control experiment, H3 K56 is acetylated by Rtt109p and incorporated into chromatin during S phase; the H3 K56 acetyl groups are removed by Hst3p and Hst4p activity in G2, M and the following G1 so chromosomes begin the second cell cycle with unacetylated H3 K56 in chromatin. In experimental condition #1 the NAM treatment was present during the first cell division and removed when cells were released from nocodazole. The results suggest that H3 K56 was deacetylated before entry into the second S phase. In experimental condition #2 NAM was present for the whole time course. **b** Effect of 50 mM NAM on a strain carrying wild-type histone H3 genes (YJT503) and a strain expressing an H3 K56R substitution (YIC339) as the sole source of histone H3. **c** 5ORIΔ-ΔR loss rate determined as the fraction of half-sectored colonies. Cells in the control culture were synchronized with α-factor, then released into medium containing nocodazole and incubated for 3 h. Samples were collected from the exponentially growing culture (exp), at the α-factor block (G1), at the nocodazole block (1st cell cycle) and 3 h after nocodazole release (2nd cell cycle). The two experimental cultures were released from the α-factor block into medium containing nocodazole +50 mM NAM for 3 h (1st cell cycle). The nocodazole block was removed by washing the cells and resuspending them in YPD (expt. #1) or YPD containing 50 mM NAM (expt. #2); samples were collected after 3 h (2nd cell cycle). Samples from each time point were diluted and plated. Plates were incubated for 5 days at 30 °C and scored for half-sectored colonies. Loss rates were calculated as the fraction of half-sectored colonies. The *graph* shows mean ± SD, calculated from at least 4 different replicas. **d** FACS analysis

To determine at what point in the cell cycle failure to deacylate H3K56Ac causes the 5ORIΔ-ΔR fragment to become unstable, we made use of a potent non-competitive sirtuin inhibitor, nicotinamide (NAM) (Bitterman et al. 2002), to temporarily inactivate sirtuins, including Hst3p and Hst4p, in a wild-type strain. We found that 50 mM NAM is sufficient to destabilize the 5ORIΔ-ΔR fragment in an *HST3* background (Fig. 4b), and that it causes H3 K56 hyperacetylation levels comparable to* hst3*Δ mutants, as shown by Western blotting using a specific anti-H3K56Ac antibody (Figure S3). Moreover, we showed that 50 mM NAM did not cause increased sectoring in the strain expressing H3K56R as the sole source of histone H3, demonstrating that Hst3p and Hst4p are the crucial targets of NAM inhibition in 5ORIΔ-ΔR fragment maintenance (Fig. 4b). This finding is consistent with the observation that Hst3p and Hst4p are the HDACs for H3K56Ac and that other single or multiple sirtuin deletions do not affect H3K56Ac levels (Celic et al. 2006).

We followed cells treated with NAM during two consecutive cell cycles, and monitored the loss rate of the 5ORIΔ-ΔR fragment as the proportion of half-sectored colonies, which result from chromosome loss events occurring in the first division of a cell that forms a colony. In control experiment, we showed that neither α-factor synchronization nor nocodazole treatment caused instability of the 5ORIΔ-ΔR fragment relative to the untreated culture (Fig. 4c, control).

In the experimental cultures, we expected to see no increase in the loss rate of the 5ORIΔ-ΔR fragment during the first cell division following treatment with NAM because cells accumulate H3K56Ac only in newly assembled chromatin behind the replication fork. However, we expected a dramatic increase in the loss rate of the 5ORIΔ-ΔR fragment during the second cell division, because cells had to replicate chromatin marked with H3 K56Ac during the second S phase. Indeed that was the result (Fig. 4).

Experimental cultures were treated with NAM either for the 1st cell cycle (expt. 1) or both 1st and 2nd cell cycle (expt. 2). We expected to see an increase in loss rate of the 5ORIΔ-ΔR fragment in the second division of both experimental conditions; however, only when cultures were left in the presence of NAM during both cell cycles did they show a significant increase in the loss rate of the 5ORIΔ-ΔR fragment 2.1 ± 0.5 × 10^−2^ (Fig. 4c, experiment 2). A likely explanation is that expression of Hst4p as cells recovered from the nocodazole block and during the subsequent G1 (Maas et al. 2006) allowed deacetylation of H3 K56Ac in the absence of NAM in experiment 1. Overall, these results are consistent with the hypothesis that H3K56Ac is detrimental for the maintenance of the 5ORIΔ-ΔR fragment only when cells enter S phase in the presence of H3K56Ac-containing chromatin, and indicate that is replication of H3K56Ac-containing chromatin that causes chromosome loss.

### DNA replication initiation is not impaired on the 5ORIΔ-ΔR fragment in the *hst3* mutant

**Fig. 5:**
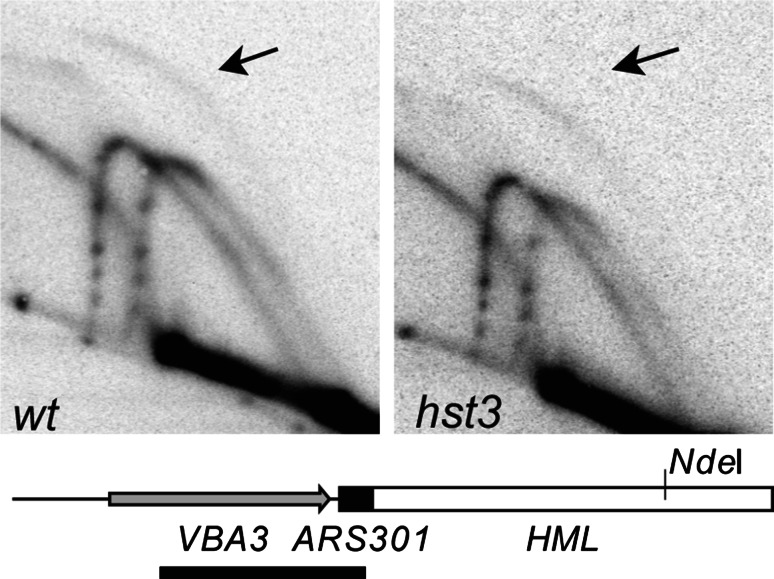
Activity of dormant origin *ARS301* in *hst3* mutant. Genomic DNA was prepared from* hst3*Δ (YIC281) and wild-type (YJT503) strains. After *Nde*I digestion, replication intermediates were separated by 2D electrophoresis, blotted and probed with a fragment containing *VBA3* and *ARS301*, indicated by the *black bar* below the diagram of the *Nde*I fragment containing *ARS301*. The fragment from the balancer chromosome III is 4.8 kb; the extra *Nde*I site present on the 5ORIΔ-ΔR fragment shortens the *ARS301* fragment to 4.1 kb. The *ARS301* probe also hybridized to a 7.1-kb NdeI fragment on chromosome XI containing the *VBA5* gene. The *arrows* point to the *bubble-shaped* intermediates arising from the 5ORIΔ-ΔR fragment

Our previous analysis of the stabilities of various ORIΔ derivatives of chromosome III in the *ofm6*-*1* mutant suggested that this *hst3* allele causes a fork progression rather than an initiation defect during 5ORIΔ-ΔR fragment replication (Theis et al. 2007). We showed that initiation at efficient replicators, *ARS305* and *ARS315* on the balancer chromosome III, and *ARS1421*, was unaffected in the *hst3* mutant. Moreover, we could not detect a decrease in mitotic stability of a plasmid carrying multiple ARS elements relative to a plasmid a single copy of *ARS1* indicating that replication initiation at *ARS1* is not affected. We used a restriction-site polymorphism present near *ARS301* to distinguish the replication intermediates arising from *ARS301* on the balancer chromosome III from those arising from *ARS301* on the 5ORIΔ-ΔR fragment. We found bubble-shaped intermediates arising from the fragment but not from the balancer chromosome III, indicating that *ARS301* fires only in the 5ORIΔ-ΔR chromosome (Theis et al. 2007). However, we had not tested the possibility that activation of the dormant origins, still present on the left arm of the 5ORIΔ-ΔR fragment, is defective in an *hst3* strain. To this end, we tested *ARS301* activity on the 5ORIΔ-ΔR fragment in the *hst3* mutant (Fig. 5). The presence of bubble-shaped intermediates in the pattern arising from the 5ORIΔ-ΔR fragment and not in the pattern from the balancer chromosome III confirmed that DNA replication initiates at *ARS301* on the 5ORIΔ-ΔR fragment in both* hst3*Δ and *HST3* strains. To compare the relative intensities of the bubble arcs in the pattern from *HST3* and* hst3*Δ strains, we calculated the ratio of signal in the bubble arc to signal in the ascending portion of the Y-arc produced by the 5ORIΔ-ΔR fragment in each strain. In the gel in Fig. 5, the ratio was 0.52 in the wild-type *HST3* strain and 0.9 in the* hst3*Δ mutant. In a second experiment, we obtained similar values, 0.75 in the wild-type *HST3* strain and 0.62 in the* hst3*Δ mutant. We conclude that initiation at the dormant origins present on the 5ORIΔ-ΔR fragment is not defective in the* hst3*Δ strain. Together with our previous analysis of efficient origins, these data indicate that replication initiation at the canonical ARS elements analyzed is not affected by the* hst3*Δ mutation, although the 2D gel technique we used may not be sensitive enough to detect a subtle defect. We conclude that *hst3∆* primarily causes a fork progression defect. We cannot rule out the possibility that this fork progression defect represents a failure to activate dormant origins that have not been identified by the canonical ARS assay yet would rescue the replication of natural chromosomes in the case of double fork-stall events.

## Discussion

This work showed that the NAD^+^-dependent histone deacetylase encoded by *HST3* plays an important role in maintenance of the 5ORIΔ-ΔR fragment of *S. cerevisiae* chromosome III. These results suggest that Hst3p is important for the replication of wild-type chromosomes that have long gaps between replication origins that result from stochastic initiation events. While others have shown the production of gross chromosomal rearrangements and other spontaneous mutations in *hst3hst4* double mutants, we explored more directly how DNA replication is affected in *hst3* mutants and we established a link between replication of a long interorigin gap and H3 K56 acetylation.

Identification of *ofm6*-*1* as an allele of *HST3* required a combination of approaches: a genome-wide analysis of SNPs present in the mutant and not in the wild-type parent, a genome-wide screening of the yeast viable deletion collection for new Ofm mutants (Theis et al. 2010) and classical genetic analysis. Using these approaches, we identified a single base-pair change that creates a nonsense codon in the *HST3* open reading frame as the *ofm6*-*1* mutation. The Ofm phenotype is the first report of a chromosome instability phenotype of an *hst3* single mutant.

We showed, for the first time, that despite substantial difference in amino acid sequence and likely differences in the regulation of protein stability (Delgoshaie et al. 2014; Edenberg et al. 2014), Hst4p could fully substitute for HST3p when expressed under the HST3 promoter. This result indicates that either the timing or level of expression of Hst3p is important for 5ORIΔ-ΔR chromosome maintenance, not differences in amino acid sequence between Hst3p and Hst4p.

Moreover, using strains expressing derivatives of histone H3 in which K56 was changed either to glutamine (K56Q) or arginine (K56R) as the sole source of histone H3, and then following 5ORIΔ-ΔR fragment stability in both *HST3* and* hst3*Δ strains we showed that H3K56Ac is the Hst3p substrate critical for chromosome maintenance.

It has been shown that H3K56 acetyl groups are transiently present in chromatin, being incorporated in new nucleosomes following the passage of the replication fork and removed prior to the next S phase (Masumoto et al. 2005). The removal of acetyl groups on H3K56 is important because constitutive H3 K56 hyperacetylation, as in the* hst3*Δ* hst4*Δ double mutant, causes defects in cell cycle progression, increased chromosome loss, increased spontaneous DNA damage, acute sensitivity to genotoxic agents, and thermosensitivity (Celic et al. 2006; Maas et al. 2006; Thaminy et al. 2007). Following cells during two consecutive cell cycles and monitoring the chromosome loss in the presence of NAM, a non-competitive sirtuin inhibitor, we showed a dramatic increase in the loss rate of the 5ORIΔ-fragment during the second cell division. These data confirm the importance of removing the H3K56Ac mark from chromatin before cells enter into the following cell cycle, as it appears that the presence of H3K56Ac on chromatin when cells enter S phase is detrimental for chromosome maintenance.

We confirmed that components of the H3 K56 acetylation pathway are involved in chromosome maintenance as shown by our findings that deletion of any one of the genes, *RTT109*, *RTT101*, *MMS22*, or *ASF1*, suppressed the* hst3*Δ Ofm phenotype. The problems caused by inappropriate H3K56 acetylation in the *hst3* mutant can be alleviated in two ways. The first way is preventing H3K56 acetylation, either by deleting the acetyltransferase, Rtt109p, or by deleting the chaperone required for Rtt109p activity, Asf1p. The second way is by preventing H3K56Ac from being incorporated into chromatin by deleting a component of the Rtt101p-Mms1p-Mms22p ubiquitin ligase responsible for releasing the H3K56Ac-H4 histone heterodimer from the Rtt109p/Asf1p complex and transferring it to other chaperones, CAF1 and Rtt106p, for deposition into chromatin (Han et al. 2013; Zaidi et al. 2008).

It is not clear how the acetylation of H3K56 contributes to the chromosome loss phenotype. We propose that, in the absence of Hst3p, which is expressed earlier in the cell cycle and at higher levels than Hst4p (Maas et al. 2006), residual H3K56Ac is left on chromatin after Hst4p is degraded during mitosis, causing chromosomes to enter the next cell cycle with H3K56Ac remaining in their chromatin. The 5ORIΔ-ΔR fragment may be particularly vulnerable to retention of this mark because at least a fraction of 5ORIΔ-ΔR fragments are late replicating for two reasons. First, our single molecule analysis of the 5ORIΔ-ΔR fragments indicates that replication initiates at only one or two places in each molecule in wild-type cells, causing replication forks to traverse longer-than-normal distances, thereby delaying completion of replication (Wang et al. manuscript in preparation). Second, a fraction of 5ORIΔ-ΔR fragments probably initiate replication later than most chromosomes, as we have shown that ‘dormant’ replicators on the left end of the 5ORIΔ-ΔR fragment are activated in about 50 % of the population (Dershowitz et al. 2007), and these replicators appear to be programmed to initiate late (Santocanale et al. 1999). Thus, one explanation for the instability of the 5ORIΔ-ΔR fragment in *hst3* mutants is that in a small fraction of cells the fragment is extremely late replicating and enters the next cell cycle carrying the H3 K56Ac mark; the presence of this mark perturbs the replication of the fragment in the subsequent S phase by causing a replication fork progression defect. While there are almost certainly other late replicating regions of the genome, including the tandem array of rDNA genes (Dulev et al. 2009; Pasero et al. 2002), chromosomes with a normal complement of replicators are less sensitive than the 5ORIΔ-ΔR fragment to the replisome perturbation caused by H3K56Ac because forks from adjacent replicators are available to replicate regions adjacent to stalled or collapsed forks.

A key question is why forks stall and chromosomes are lost when replication forks have to move through chromatin carrying the H3K56Ac mark. Recent work suggests two possibilities. The first is based on the previous observation that abnormal persistence of the H3K56Ac mark affects replisome stability or movement (Celic et al. 2008). Overexpression of *RFC1*, which encodes a subunit of the complex that loads PCNA, the Polδ and Polε processivity factor, suppresses the temperature‐sensitive growth defect and the sensitivity to genotoxic agents of the *hst3 hst4* double mutant. The high levels of H3 K56Ac are not altered in the strains overexpressing *RFC1*, indicating that increasing the levels of Rfc1p allows cells to survive in the presence of H3 K56 hyperacetylation. Moreover, mutant derivatives of replisome components, including a truncated derivative of DNA Polε encoded by *pol2‐11* and epitope‐tagged derivatives of PCNA and Cdc45p that support growth of *HST3 HST4* strains, are lethal in combination with* hst3*Δ* hst4*Δ, providing further evidence that replisomes are stressed in *hst3 hst4* strains. Transient treatment of *hst3hst4* strains with low levels of MMS, an alkylating agent that alkylates adenine to 3-methyl adenine, which blocks DNA polymerases, or hydroxyurea, which inhibits ribonucleotide reductase and starves cells for deoxynucleotides for DNA synthesis, causes loss of viability and delays in completion of S phase (Simoneau et al. 2015). Cells activate the DNA damage response (DDR), which normally stabilizes replisomes and delays cell cycle progression (Labib and De Piccoli 2011) and die with incompletely replicated chromosomes, implying that impeding replication during a single S phase is sufficient to kill the double mutant.

Interestingly, deletion of *CTF4* suppressed both the temperature sensitivity and HU sensitivity of the* hst3*Δ* hst4*Δ double mutant (Celic et al. 2008). Ctf4p functions in both chromosome segregation and DNA replication (Gambus et al. 2006; Lengronne et al. 2006; Spencer et al. 1990). It is a component of the replisome that couples Polα to the MCM helicase on the lagging strand through its interaction with GINS, and the Ctf4 WD40 domain interacts with several fragments of Mms22p in a two-hybrid interaction (Gambus et al. 2006). Replisomes frequently pause at discrete natural pause sites (Ivessa et al. 2003) or sites of DNA damage. Under these conditions of replicative stress, the S phase checkpoint is activated, and the presence of H3K56Ac appears to promote the recruitment of the Rtt101p/Mms1p/Mms22p ubiquitin ligase via the interaction of the Mms22 protein with Ctf4p trimer. Ubiquitination of either Ctf4p or some other component of the replisome results in uncoupling the helicase from Polα, leading to regions of single-stranded DNA and inactivating the replisome (Gambus et al. 2009; Tanaka et al. 2009). If loss of the 5ORI∆ fragment in the *hst3* mutant is caused by destabilization of stalled replisomes, we would predict that deletion of *ctf4* would suppress the *hst3* mutation.

The second possibility is based on the finding that (Simoneau et al. 2015; Wurtele et al. 2012) point mutations in two other histones that reduce levels of H4K14Ac or H3K79Me reduced both the temperature sensitivity and MMS sensitivity of the *hst3hst4* double mutant without reducing H3K56Ac levels (Simoneau et al. 2015). These modifications are abundant in yeast chromatin, and deletion of the genes encoding proteins that catalyze these modifications also partially suppressed the phenotypes of the *hst3∆hst4∆* mutant. The H3K79Me modification contributes to the activation of the DDR kinase, Rad53p, in the double mutant by recruiting Rad9p to chromatin, where it mediates the activation of Rad53p. A *rad9∆* mutation partially suppressed the phenotype of the *hst3∆hst4∆* mutant. In contrast to this result, we have found that a *rad9∆* causes increased loss of the 5ORI∆ chromosome, and have also argued that DNA damage is unlikely to cause the loss of this chromosome (Theis et al. 2010). Examining the phenotypes expressing H4k14R or H3K79R would provide a further test of this idea.

Overall, the surprising stability of the ORI∆ chromosome shows that even though origin distribution tends to be even throughout the genome to minimize fork collapse, cells can tolerate long interorigin gaps. However, those chromosomes become more sensitive to perturbation of the replication machinery, limiting licensing factors, DNA checkpoints and, as we show proper turnover of histone acetylation.

## Electronic supplementary material


**Figure S1** Identification of a point mutation in *HST3* using tiling microarray data and the SNPScanner algorithm. The likelihood that each nucleotide site is polymorphic, as compared with the S288c reference genome, was computed and compared for wildtype (green) and ofm6-1 mutant (yellow). A mutation was predicted at nucleotide 378,826 (yellow arrow) and confirmed by Sanger sequencing analysis (TIFF 1406 kb)

**Figure S2** Unique mutations are not detected in the *ofm6-1* mutant (yellow) in *BUB3* or *IRC12*/*YOR024W* (TIFF 1717 kb)

**Figure S3 Panel A** Wild type (strain name), *ofm6-1*, *hst3* and *cdc15* were synchronized with alpha factor and release into nocodazole. Samples were collected at the alpha factor block (G1 cells), after 40 minutes after the block release (S phase cells) and at the nocodazole block (G2 cells). Cell cycle progression was monitored by FACS. Protein were extracted and run on an SDS-page gel, the blots were probed with both anti-H3 antibody and anti H3 K56 Ac. As expected in the wild type backgrond the H3 K56 acetyl signal is low, almost blank in G1 blocked cells, it gets incorporated during DNA synthesis and removed in G2. In the two *hst3* isolates *ofm6-1* and* hst3*Δ the H3K56 Ac signal remain strong in G2, suggesting that the acetyl group is not removed in the mutant. **Panel B** Wild type (strain name),* rtt109*Δ and *ofm6-1* mutant were synchronized with alpha factor and release into nocodazole with and without nicodinammide. Samples were collected at the alpha factor block (alpha), after 40 minutes after the block release (40 minutes) and at the nocodazole block (100 minutes). Cell cycle progression was monitored by FACS. Treatment of the wild type with NAM causes accumulation of the h3 K56 acetylation in nocodazole blocked cells, suggesting it is recapitulating an *hst3* phenotype (TIFF 7341 kb)

**Table S1** (DOC 95 kb)

**Table S2** (DOC 35 kb)
