# Supplementary figures and images for: Hst3p, a histone deacetylase, promotes maintenance of Saccharomyces cerevisiae chromosome III lacking efficient replication origins
Source: Mol Genet Genomics. 2015 Aug 29;291:271–83. doi: 10.1007/s00438-015-1105-8 (PMC4729790; doi:10.1007/s00438-015-1105-8)

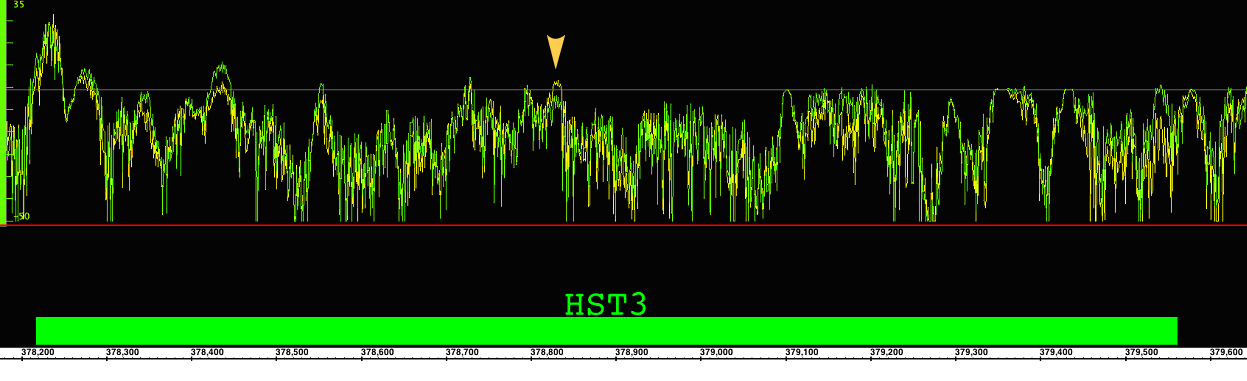

Supplement: Supplementary file 1 — Figure S1 Identification of a point mutation in HST3 using tiling microarray data and the SNPScanner algorithm. The likelihood that each nucleotide site is polymorphic, as compared with the S288c reference genome, was computed and compared for wildtype (green) and ofm6-1 mutant (yellow). A mutation was predicted at nucleotide 378,826 (yellow arrow) and confirmed by Sanger sequencing analysis (TIFF 1406 kb) [file 438_2015_1105_MOESM1_ESM.tif]

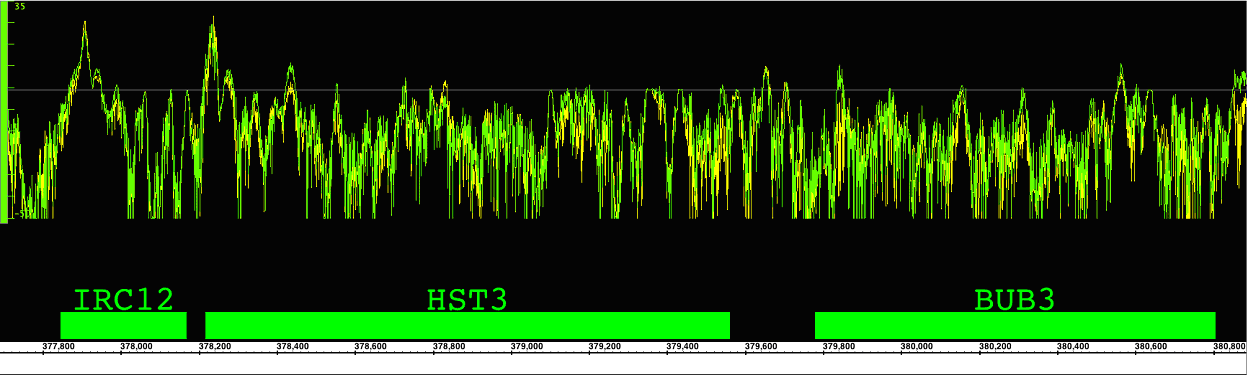

Supplement: Supplementary file 2 — Figure S2 Unique mutations are not detected in the ofm6-1 mutant (yellow) in BUB3 or IRC12/YOR024W (TIFF 1717 kb) [file 438_2015_1105_MOESM2_ESM.tif]

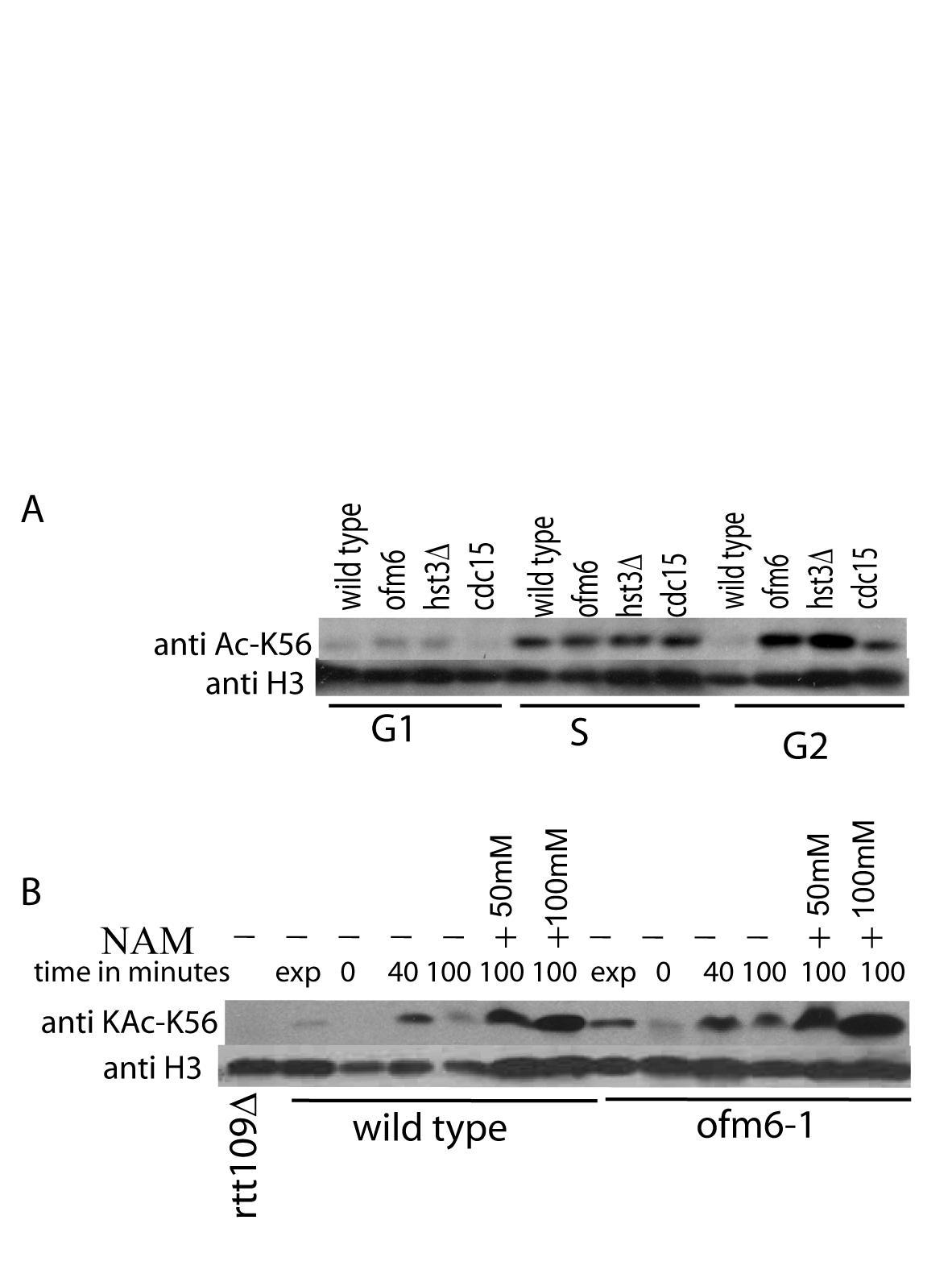

Supplement: Supplementary file 3 — Figure S3 Panel A Wild type (strain name), ofm6-1, hst3 and cdc15 were synchronized with alpha factor and release into nocodazole. Samples were collected at the alpha factor block (G1 cells), after 40 minutes after the block release (S phase cells) and at the nocodazole block (G2 cells). Cell cycle progression was monitored by FACS. Protein were extracted and run on an SDS-page gel, the blots were probed with both anti-H3 antibody and anti H3 K56 Ac. As expected in the wild type backgrond the H3 K56 acetyl signal is low, almost blank in G1 blocked cells, it gets incorporated during DNA synthesis and removed in G2. In the two hst3 isolates ofm6-1 and hst3Δ the H3K56 Ac signal remain strong in G2, suggesting that the acetyl group is not removed in the mutant. Panel B Wild type (strain name), rtt109Δ and ofm6-1 mutant were synchronized with alpha factor and release into nocodazole with and without nicodinammide. Samples were collected at the alpha factor block (alpha), after 40 minutes after the block release (40 minutes) and at the nocodazole block (100 minutes). Cell cycle progression was monitored by FACS. Treatment of the wild type with NAM causes accumulation of the h3 K56 acetylation in nocodazole blocked cells, suggesting it is recapitulating an hst3 phenotype (TIFF 7341 kb) [file 438_2015_1105_MOESM3_ESM.tif]
