# Supplementary material for: Hst3p, a histone deacetylase, promotes maintenance of Saccharomyces cerevisiae chromosome III lacking efficient replication origins
Source: Mol Genet Genomics. 2015 Aug 29;291:271–83. doi: 10.1007/s00438-015-1105-8 (PMC4729790; doi:10.1007/s00438-015-1105-8)
Supplement: Supplementary file 4 — Table S1 (DOC 95 kb) [file 438_2015_1105_MOESM4_ESM.doc]

Table S1

| **Strain name Genotype Source** | | |
| --- | --- | --- |
| YKN10, YKN15 | *MAT****a*** *HIS4 leu2- Δ1*  *his4-280 LEU2 C2G::ADE2 H9G::TRP1 Tel 5ORIΔ* *ura3-52 lys2-801 trp1-Δ63 his3-Δ200 ade2-101 cyh2 can1 kar1-Δ15 ARO7* | This work |
| YJT503 | *MAT****a*** *HIS4 leu2- Δ1*  *ura3-52 lys2-801 trp1-Δ63 his3Δ ade2-101 cyh2 can1 kar1-Δ15 ARO7 bar1Δ* | This work |
| YJT3 | *MATα leu2- Δ1*  *ura3-52 lys2-801 trp1-Δ63 HIS ade2-101 cyh2 can1 kar1-Δ15 aro7Δ::kanMX* | (Theis, Dershowitz et al. 2007) |
| F510α4A1-4 | *MATα his4-290 LEU2*  *his4-280 LEU2 C2G::SUP11-1 H9G::TRP1 Tel 0ORIΔ*  *ura3-52 trp1-Δ63 ade2-101* | (Theis, Dershowitz et al. 2007) |
| F013αB2C-1C | *MATα his4-290 LEU2 __*  *his4-280 LEU2 C2G::SUP11-1 H9G::TRP1 Tel 0ORIΔ*  *ura3-52 trp1-Δ63 ade2-101* *kar1-Δ15* | (Theis, Dershowitz et al. 2007) |
| YJT417 | YKN15 *ofm6-1* | This work |
| YIC247 | YKN15 *hst3Δ::kanMX* | This work |
| YIC257 | YKN15 *ofm6-1* (reconstructed point mutant) | This work |
| YIC281 | YKN15 *hst3Δ::HIS3* | This work |
| YIC331, YIC332, YIC333, YIC334 | YKN15 *hst3Δ::HST3pr-HST4* | This work |
| YIC271 | YKN15 *ofm6-1 ygl119w::HST3* | This work |
| YIC273 | YKN15 *hst3Δ::kanMX* *ygl119w::HST3* | This work |
| YIC275 | YKN15 *ofm6-1 ygl119w::HST3* | This work |
| YIC263 | YKN15 *rtt109Δ::kanMX* | This work |
| YIC260 | YKN15 *ofm6-1 rtt109Δ::kanMX* | This work |
| YIC290 | YJT503 *asf1Δ::kanMX* | This work |
| YIC296 | YKN15 *hst3Δ::HIS3 asf1Δ::kanMX* | This work |
| YIC302 | YKN15 vps75Δ::*kanMX* | This work |
| YIC301 | YKN15 *ofm6-1 vps75Δ::kanMX* | This work |
| YIC304 | YJT503 *mms22Δ::kanMX* | This work |
| YIC306 | YKN15 *hst3Δ::HIS3* *mms22Δ::kanMX* | This work |
| YIC266 | YKN15 *rtt101Δ::kanMX* | This work |
| YIC264 | YKN15 *ofm6-1 rtt101Δ::kanMX* | This work |
| YJT371 | *Matα can1Δ::STE2pr-HIS3 lyp1Δ his3Δ1 leu2 ura3 LYS2 ade2::NAT1 TRP1 KAR1 ARO7 cyh2 ofm6-1* | This work |
| BY4741 | *MATa his3-Δ1 leuΔ0 lys2-Δ0 ura3-Δ0* | Open Biosystems |
| yel030wΔ | BY4741 *yel030w*Δ*::kanMX* | Open Biosystems |
| ygl010wΔ | BY4741 *ygl010w*Δ*::kanMX* | Open Biosystems |
| ylr001cΔ | BY4741 *ylr001c*Δ*::kanMX* | Open Biosystems |
| ydl012cΔ | BY4741 *ydl012c*Δ*::kanMX* | Open Biosystems |
| ymr002wΔ | BY4741 *ymr002w*Δ*::kanMX* | Open Biosystems |
| yfr009wΔ | BY4741 *yfr009w*Δ*::kanMX* | Open Biosystems |
| yhl002wΔ | BY4741 *yhl002w*Δ*::kanMX* | Open Biosystems |
| yal007cΔ | BY4741 *yal007c*Δ*::kanMX* | Open Biosystems |
| ybl019wΔ | BY4741 *ybl019w*Δ*::kanMX* | Open Biosystems |
| yir004wΔ | BY4741 *yir004*wΔ*::kanMX* | Open Biosystems |
| yjl007cΔ | BY4741 *yjl007c*Δ*::kanMX* | Open Biosystems |
| ykr005cΔ | BY4741 *ykr005c*Δ*::kanMX* | Open Biosystems |
| ynl004wΔ | BY4741 *ynl004w*Δ*::kanMX* | Open Biosystems |
| ynl009wΔ | BY4741 *ynl009w*Δ*::kanMX* | Open Biosystems |
| yol002cΔ | BY4741 *yol002cΔ::kanMX* | Open Biosystems |
| yor003wΔ | BY4741 *yor003w*Δ*::kanMX* | Open Biosystems |
| yor025wΔ | BY4741 *hst3*Δ*::kanMX* | Open Biosystems |
| MSY421 | *MATα trp1-289 ura3-53 his3-1 leu2-3,112 (hht2-hhf2)Δ (hht1-hhf1)Δ pMS329-HHT1-HHF1-CEN-URA3* | M.M. Smith |
| YIC335 | *MATα his4-290 LEU2*    *his4-280 LEU2 C2G::ADE2 H9G::TRP1 Tel 5ORIΔ*  *ura3-52 LYS ade2-101 trp1-Δ63 can1 cyh2 his ARO (hht2-hhf2)Δ (hht1-hhf1)Δ*  *pMS329-HHT1-HHF1-CEN-URA3* | This work |
| YIC339 | *MATα his4-290 LEU2*    *his4-280 LEU2 C2G::ADE2 H9G::TRP1 Tel 5ORIΔ*  *ura3-52 LYS ade2-101 trp1-Δ63 can1 cyh2 his ARO (hht2-hhf2)Δ (hht1-hhf1)Δ*  *phht2K56R-HHF2-CEN-HIS3* | This work |
| YIC341 | *MATα his4-290 LEU2*    *his4-280 LEU2 C2G::ADE2 H9G::TRP1 Tel 5ORIΔ*  *ura3-52 LYS ade2-101 trp1-Δ63 can1 cyh2 his ARO (hht2-hhf2)Δ (hht1-hhf1)Δ*  *phht2K56Q-HHF2-CEN-HIS3* | This work |
| YIC347 | *MATα his4-290 LEU2*    *his4-280 LEU2 C2G::ADE2 H9G::TRP1 Tel 5ORIΔ*  *ura3-52 LYS ade2-101 trp1-Δ63 can1 cyh2 his ARO (hht2-hhf2)Δ (hht1-hhf1)Δ*  *hst3Δ::kanMX pMS329-HHT1-HHF1-CEN-URA3* | This work |
| YIC349 | *MATα his4-290 LEU2*    *his4-280 LEU2 C2G::ADE2 H9G::TRP1 Tel 5ORIΔ*  *ura3-52 LYS ade2-101 trp1-Δ63 can1 cyh2 his ARO (hht2-hhf2)Δ (hht1-hhf1)Δ*  *hst3Δ::kanMX phht2K56R-HHF2-CEN-HIS3* | This work |
| YIC350 | *MATα his4-290 LEU2*    *his4-280 LEU2 C2G::ADE2 H9G::TRP1 Tel 5ORIΔ*  *ura3-52 LYS ade2-101 trp1-Δ63 can1 cyh2 his ARO (hht2-hhf2)Δ (hht1-hhf1)Δ*  *hst3Δ::kanMX phht2K56Q-HHF2-CEN-HIS3* | This work |

Theis, J. F., A. Dershowitz, et al. (2007). "Identification of mutations that decrease the stability of a fragment of Saccharomyces cerevisiae chromosome III lacking efficient replicators." Genetics **177**(3): 1445-58.
