# Supplementary material for: Hst3p, a histone deacetylase, promotes maintenance of Saccharomyces cerevisiae chromosome III lacking efficient replication origins
Source: Mol Genet Genomics. 2015 Aug 29;291:271–83. doi: 10.1007/s00438-015-1105-8 (PMC4729790; doi:10.1007/s00438-015-1105-8)
Supplement: Supplementary file 5 — Table S2 (DOC 35 kb) [file 438_2015_1105_MOESM5_ESM.doc]

| **Centromere-linked deletion and**  **chromosome location** | **PD** | **NPD** | **TT** |
| --- | --- | --- | --- |
| *yal007cΔ*−chrI | 5 | 9 | 1 |
| *ybl019wΔ*−chrII | 4 | 2 | 1 |
| *ydl012cΔ*−chrIV | 6 | 5 | 1 |
| *yel030wΔ*−chrV | 5 | 2 | 0 |
| *yfr009wΔ*-chrVI | 5 | 6 | 0 |
| *ygl010wΔ*−chrVII | 3 | 3 | 0 |
| *yhl002wΔ*−chrVIII | 8 | 5 | 1 |
| *yir004wΔ*−chrIX | 2 | 6 | 0 |
| *yjl007cΔ*−chrX | 4 | 2 | 0 |
| *ykr005cΔ*−chrXI | 6 | 3 | 0 |
| *ylr001cΔ*−chrXII | 6 | 8 | 3 |
| *ymr002wΔ*−chrXIII | 10 | 8 | 0 |
| *ynl004wΔ*,  *ynl009wΔ*−chrXIV | 10 | 5 | 1 |
| *yol002cΔ*, *yor003wΔ*−chrXV | 12 | 0 | 2 |

**Table S2 Centromere linkage analysis**

The strains carrying the *kanMX*-marked deletions crossed to *ofm6-1* (YJT371) in the centromere linkage analysis are listed in the first column. The last three columns show the distribution of tetrads that fell in PD (Parental di-type), NPD (non-parental di-type) and TT (tetra-type) classes for each cross.
